# The effects of psychosocial interventions using generic photos on social interaction, mood and quality of life of persons with dementia: a systematic review

**DOI:** 10.1186/s12877-023-04270-w

**Published:** 2023-09-14

**Authors:** Josephine Rose Orejana Tan, Petra Boersma, Teake P. Ettema, Caroline H. M. Planting, Soraya Clark, Robbert J. J. Gobbens, Rose-Marie Dröes

**Affiliations:** 1 Department of Psychiatry, Amsterdam University Medical Centers, Location VUmc / Amsterdam Public Health Research Institute, Amsterdam, The Netherlands; 2 Faculty of Health, Sports and Social Work, Inholland University of Applied Sciences, Amsterdam, The Netherlands; 3 Ben Sajet Centrum, Amsterdam, the Netherlands; 4 GGZ in Geest, Medical Information Specialist, Amsterdam, The Netherlands; 5 Zonnehuisgroep Amstelland, Amstelveen, The Netherlands; 6 Department of Family Medicine and Population Health, Faculty of Medicine and Health Sciences, University of Antwerp, Antwerp, Belgium; 7 Tranzo, Tilburg University, Tilburg, the Netherlands

**Keywords:** Generic photos, Art interventions, Dementia, Social interaction, Mood, Quality of life

## Abstract

**Background:**

Although family photos are often used in the psychosocial care for people with dementia, little is known about the use and effectiveness of generic photos. This systematic literature review explored psychosocial interventions using generic photos for people with dementia, and the effects they have on their social interaction and/or mood and/or quality of life. In addition, it was investigated whether these interventions made use of technology in its implementation.

**Methods:**

A systematic search on the following databases was performed: PubMed, Embase, APA PsychInfo, Cinahl, Web of Science, Scopus and Cochrane Central. Inclusion and exclusion criteria were based on the PICO model (Population, Intervention, Comparison, Outcome), and quality assessment was undertaken using the Weight of Evidence Framework. Narrative synthesis was undertaken to summarize study characteristics- settings and designs, type of psychosocial interventions identified, type of photos and technology used, outcome measures, and results.

**Results:**

A total of 2,035 results were found, however after title, abstract and full-text screening, a total of 8 studies were included. The most common psychosocial intervention using generic photos was found to be reminiscence therapy, followed by art-viewing activities. In studies that used technology, it was reported that viewing digitalized photos were either similar or better to conventional printed photos. Despite photos being generic, it was found that generic photos could still hold personal significance to the person with dementia. Some positive and significant effects were found for the outcomes social interaction, mood and quality of life, though no study evaluated all three outcomes. Two studies were rated as having high overall quality, 4 were rated as fair, and 2 studies had a low quality assessment rating.

**Conclusion:**

Studies found using generic photos were limited, showing varying outcomes and methodological quality. Firm conclusions on the effectiveness of interventions using generic photos are not possible. However, the use of generic photos in psychosocial interventions is a promising area for future research. Researchers should consider studies with better methodological quality and larger samples; and qualitative studies where the intention is to get better insight into successful implementation and impact mechanisms of such psychosocial interventions.

**Trial registration:**

n/a.

**Supplementary Information:**

The online version contains supplementary material available at 10.1186/s12877-023-04270-w.

## Background

Psychosocial interventions for people with dementia have been known to incorporate the arts in many forms, such as art-making involving painting, taking photos, drawing, pottery or art-viewing involving interactive tours in museums, for example [1–3]. Art has gained acceptance as an effective tool to promote health, to cultivate social relationships, and to provide mental stimulation as well as independence [4, 5]. This aspect of social participation in art therapy is consistent with the INTERDEM Social Health Taskforce operationalization of the concept of social health of people with dementia, particularly the third dimension, ‘participation in social activities’ [6]. Art therapies or activities involving art provide a way for people living with dementia, to still experience meaningful interactions with their social network, improved quality of life as well as improved sense of pleasure or mood [1, 2]. Art based activities, as recommended by the INTERDEM Social Health Taskforce, can also be easily adapted to fit the person’s individual needs [6].

Integrated art therapies (such as performing arts, visual arts, design and craft, and even online digital and electronic arts), can use a variety of props or materials (textiles, photography, painting, animations, or computer graphics), and are still considered a less costly option compared to medical health interventions [4]. Integrated art therapies can then potentially provide a less costly alternative, especially for people with dementia and their families living in lower income countries [7]. Furthermore, integrated art therapies can provide a culturally flexible solution to a challenge specifically faced in Europe which is the existence of numerous languages, cultures and differences in care systems, that make it difficult to smoothly deliver a new intervention from its developmental stages to broad implementation [8].

Photos in particular, have been a common material used in psychosocial interventions for persons living with dementia, most notably in reminiscence therapy, where past experiences, activities and events of the person with dementia are discussed, usually with a carer, using supporting materials to stimulate the memory [9]. Previous studies that used photos in activities (either on their own or with other elements like music) with people with dementia found that activities using photos have the potential to enhance social interaction and feelings of closeness between the person with dementia and their carer [10–12] through eliciting emotional responses brought about by sharing and reliving valued personal stories and experiences with each other [10, 13]. Activities using photos were also shown to have positive effects on mood [11, 14] and quality of life [14, 15] of people living with dementia.

Most of the studies above [10–12, 14] made use of personal photographs (i.e., family photographs for example), as they have generally been thought to provide better stimulation for the person with dementia. However a study of Astell and colleagues [16] showed that people with dementia told more stories that contained emotional and personal significance when shown generic photographs, as compared to when they were shown personal photographs [16]. Generic photographs as a tool in psychosocial activities have the potential of being more accessible, as it lessens the preparatory efforts that carers often need to do when asking families or relatives to collect personal photos [9]. In some cases generic photographs could be a good option when family photographs are not available or when the person with dementia experiences distress from not remembering details about their personal photos [16]. Viewing generic photographs together can be seen as similar to other visual art programs for people with dementia such as guided interactive museum tours, where people with dementia and their carers have the opportunity to emotionally connect over the art work in the museum [5]. However, the use of generic photographs may be easier to implement, especially for people living with a moderate to severe form of dementia, where travelling may pose more of an issue. For example, instead of physically visiting a museum, one study used photos from three London museums and collections from a painter and a photographer for an art-viewing activity on a tablet-computer [17]. Finally, as mentioned earlier, generic photographs can also be cost-effective, as they have the possibility of transcending the barriers of societal or cultural differences in the form of photographs reflecting country specific or world-wide events [16].

Despite the potential of using generic photos in psychosocial interventions for people with dementia, relatively little research seems to have been done into the effect of such interventions. However, as far as we know, to date no literature reviews are available that give insight into how generic photographs are used in psychosocial interventions and these interventions’ effectiveness on social interaction and/or mood and/or quality of life of people with dementia. Therefore, we conducted a systematic literature review to address this gap. This paper reports on the methods used in the review and the findings.

The following main questions were addressed in this systematic review:

1. What types of psychosocial interventions using generic photographs currently exist for people living with dementia that aim to improve their social interaction and/or mood and/or quality of life?
2. What are the effects of current/existing psychosocial activities/interventions using generic photographs on persons with dementia's social interaction and/or mood and/or quality of life?
3. How are the effects on quality of life and/or socia interaction and/or social health evaluated in these studies?
4. Which of the studies found using generic photographs in the intervention incorporate the use of technology (i.e. tablet, computer, smart phones)

This review focuses on the outcomes social interaction, mood and quality of life to expand on the work of the INTERDEM network in improving the social health of people living with dementia, and in order to address the research gaps identified by the Social Health task force of this network in the domain of participation in social activities [6]. More specifically this review was conducted as a state-of-the-art study within the framework of a research into the development of a photo-activity intervention to improve the social health of people living with dementia. The final sub-question was added to this review because the use of technology, especially since the Covid pandemic, is becoming increasingly relevant in the field of psychosocial care in dementia, in terms of providing meaningful and engaging activities, and promoting social interaction [18].

## Methods

The PRISMA Statement for Reporting Systematic Reviews and Meta-Analyses [19] was used to structure this systematic review (see Additional files 1 and 2).

### Search strategy

The protocol for this review was not registered or published in advanced. J.T., C.P. and R.M.D. developed the initial search strategy. C.P. is an information specialist who assisted with constructing the various search strings. Initially, the search included four key elements used in search strings- Dementia-Alzheimer, Nursing homes, Photo activity, and Outcome/effect. In the end, the search string related to Nursing homes was removed because it was decided that the focus of the review was more on the use of photos in the intervention, and not necessarily the setting of the intervention. The search element ‘Outcome/effect’ was used in the search string instead of terms relating to social interaction, mood and quality of life, in order to capture as many results as possible in the initial search. This way, publications that may have named the outcomes of interests in different ways (for example, ‘well-being’ instead of ‘quality of life’) would not have been missed. The specific outcomes of interest were used as part of the inclusion/exclusion criteria in the screening phase, where authors then discussed and came upon an agreement if a study did include one of the outcomes of interest. The search term ‘qualitative’ under the search element of ‘outcome/effect’ was included in the search string because we wanted to gain deeper insight in the qualitative results of relevant intervention studies which were published in addition to the effectiveness studies. Existing studies may have been of an exploratory nature, thus utilizing a mixed methods approach. If a relevant study was mixed methods but there were no effects found, attention was still given to the qualitative results and whether or not they showed something interesting (for example, despite an intervention not having any significant effects, the participants may have experienced the intervention as beneficial and useful). Studies that were purely qualitative were excluded in the screening process to align to the scope of the research questions, which focused on effectiveness studies. The search was conducted on 14 January 2022. An additional search update in the same databases, using the same search strings, on the 9th of June 2023 was conducted. The search strings can be viewed in the supplementary files (see Additional file 3). No specific date limits were used. The following databases were used: PubMed, Embase, APA PsycInfo (EBSCO), Cinahl (EBSCO), Web of Science (Clarivate), Scopus (Elsevier), and Cochrane Reviews & Cochrane Central Register of Controlled Trials (CENTRAL). The searches were conducted from inception of the Database using thesaurus terms (MeSH terms/ Emtree etc.) and free terms. C.P. worked on exporting the results of the search into a reference management software (EndNote) and was also responsible for de-duplicating the results before screening. Whenever a trial registry entry was encountered, J.T. attempted to find published articles related to the trial registry entry using Google Scholar or a general Google search.

### Inclusion and exclusion criteria

The inclusion and exclusion criteria were based on the PICO model [20] where a clinical question is formulated based on the Population, Intervention, Comparison, and Outcome criteria, to be able to find the most relevant answers. This formulation was agreed upon by all the authors. For this current systematic review, a study met the Population criteria if the majority of the target group were persons with dementia, living in the community or nursing home. Primary participants in the included studies should have a diagnosis of dementia or mild cognitive impairment. All types of dementia (e.g., Alzheimer’s Disease [AD], vascular, mixed, dementia with Lewy Bodies [DLB], and frontotemporal dementia) were considered. Studies were excluded if the majority of the target group did not have a diagnosis of dementia. A study met the Intervention criteria if it was an effectiveness study (controlled or randomized controlled trial [RCT]) of a psychosocial intervention using generic photos aimed at improving outcomes for persons with dementia. Generic photos were defined as photos that are not personal (i.e., no family photos or participants’ own photos). Studies were included if use of generic photos was part of a wider range of tools used in an intervention (for example, in combination with music or creating artwork using generic photos). Photos could be physical or digital. Studies were excluded if the artwork was made by the person with dementia themselves (for example, art therapy where participants create art using their own photos). Studies where photos or images mentioned are those of biological outcomes (for example, MRI scans) were also excluded. A study met the Comparison criteria if it included a control condition which could be another treatment/intervention or care as usual or no intervention. Studies that did not have a control condition were excluded. Finally, a study met the Outcome criteria if it had at least one of the following outcomes for persons with dementia: social interaction, mood and/or quality of life.

Conference proceedings were excluded if they included only abstracts. If they included full papers, the researchers looked into each paper individually. Studies included in systematic reviews that came up in the search results were also evaluated individually to see if they met the inclusion and exclusion criteria.

Only published studies in the English language were included for this review. Papers not in English were included if at least the title and abstract were provided in English.

### Study selection and data extraction

All references found from C.P.’s search were first screened by title and abstract by the authors. References were divided between three pairs of the authors (J.T. with P.B., R.M.D. with S.C., and T.E. with R.G.), where each person in the pair first screened the titles and abstracts individually, then discussed their decisions with their partner before deciding on a definitive list of titles to include. Where it was difficult to decide based on abstract, the authors referred to the full text. All review pairs reached a consensus on their screening decisions. Where systematic reviews were found, authors referred the paper to J.T. who checked the full text and the papers included in the review for studies that met the criteria.

After study selection, type of data extracted by the first author from each study included the following: study design, study setting, sample characteristics and size, experimental intervention, control intervention, outcome measures used for social interaction, mood and quality of life, type of photos (paper or digitalized), and what kind of technology was used if photos were digitalized. These were then checked by a second senior author.

### Assessment of bias / Methodological quality

To assess both generic and review-specific qualities of the selected papers, the Weight of Evidence (WoE) framework was used [21]. The assessment is broken down into four categories, WoE A, B, C and D. WoE A concerns the evidence of a paper individually and judges it according to its inherent integrity and methodological quality. In this review, the National Institute of Health (NIH) tool is used as a guide in assessing the quality and internal validity included randomized controlled studies for WoE A [21, 22]. WoE B takes into account whether the evidence presented in the paper is a good fit for the questions in the review and is therefore a review-specific criteria. WoE C, also a review-specific criteria, assesses whether the characteristics of evidence (i.e., the characteristic of the sample, the study design, or analysis) presented in the paper are within a similar context to that of the review questions and therefore easily generalizable to the review. Finally, WoE D is the overall assessment, taking into account the assessment results from WoE A through C [21].

### Data synthesis

While the scope of the papers included was narrowed down through the selection process, there was still considerable heterogeneity between the studies in terms of setting, types and structure of the interventions, and outcomes, to name a few. This review for example, considered papers that included either one or more of the following outcomes ‘social interaction’, ‘mood’, and ‘quality of life’, even if these exact terms were not used in the paper, but by definition measured a similar concept. A narrative synthesis, summarizing the findings of the selected papers, was therefore deemed appropriate for this review, as this method can also address questions centered on intervention effects through the use of textual descriptions and descriptive tables [23].

## Results

**Fig. 1:**
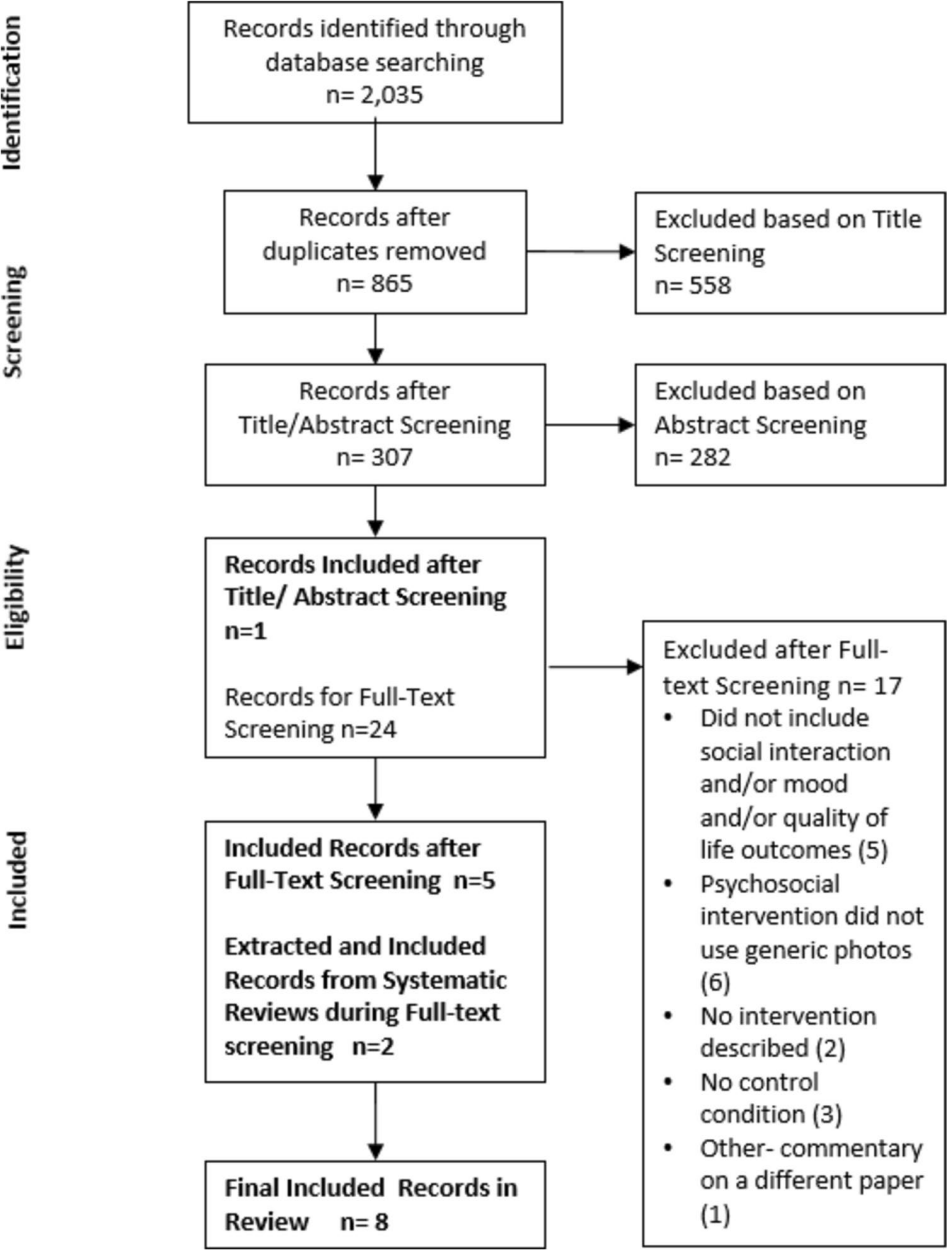
Search result screening process (PRISMA Flow Diagram)

A total of 2,035 search results were found, and after deduplication, 865 search results remained (see Fig. 1) [24]. Of the 865 search results, 130 of these were new results. One of the authors (J.T.) conducted a title/abstract screening of the new results, and following the same exclusion/inclusion criteria, found that there were no relevant articles to add to the current systematic review.

One paper was included based on abstract screening alone [25]. Twenty-four papers needed a further full-text screening, and from this, only an additional 7 papers [26–32] met the inclusion criteria, giving a total of 8 included papers. Note that [26] and [29] were papers extracted from systematic reviews that came from the original results respectively [33, 34], but were not in the final inclusion.

Seventeen papers were excluded for different reasons: Five were excluded because they did not include social interaction, mood and/or quality of life outcomes [35–39]; six were excluded because the psychosocial intervention described did not use generic photos [11, 40–43]; two were excluded because there was no intervention described [28, 44]; three were excluded due to not having a control condition [17, 45, 46]; and one was excluded [47] because upon full-text screening, it was determined that the paper was not a unique study, rather it was a commentary on another existing study [42].

### Characteristics of included studies

Table 1 describes the relevant study characteristics, including the design, a description of the setting, the sample, the experimental intervention, and the control condition. For the columns of Experimental and Control or Comparison Intervention, specific sub-sections were added to organize the information. These sub-sections are: intervention, person delivering the intervention, format, length/duration, and material. Table 2 describes the outcome measures used, whether the photos were printed or digitalized and in the latter case, the technology used. As can be seen in the tables, there is relevant heterogeneity between the studies.

**Table 1:** Characteristics of included studies

**Authors, Year, and Language of Publication**	**Study Design**	**Setting**	**Sample Characteristics and Sample Size**	**Experimental Intervention (person delivering the intervention, format, length and duration)**		**Control or Comparison Intervention (person delivering the intervention, format, length and duration)**
Aşiret & Kapucu, 2016 [26] (English)	Quasi-experimental, post-hoc	Elderly care and rehabilitation centre	Diagnosis of Alzheimer’s Disease, MMSE score from 10–24 points, 3 months in the institution, no issues with talking/communication; Average age of experimental group = 81.83 ± 4.87, Control group = 82.26 ± 5.07 *N* = 62	Intervention: Reminiscence therapy Person delivering the intervention: Investigator Format: Group sessions Length/duration: 1x/ week for 30–35 min, for 12 weeks Material: Photos of old objects were used, and personal photos requested also from participants		Intervention: Generic conversations per week (topics: religious or relevant days in the week, health of individual, or current issues) Person delivering the intervention: not mentioned Format: Group sessions Length/duration: Average of 20–25 min per week (but duration also varies by week) Material: none
Azcurra, 2012 [29] (English)	Single-blinded, parallel groups with 3 arms (intervention, active, and passive control) RCT	Nursing home;	Diagnosis of Alzheimer’s disease, according to DSM-IV, able to communicate with Holden Communication Scale score > 25, and has MMSE score above 10; Age of experimental group = 85.3 ± 5.6, Age of Active control group = 86.4 ± 4.9, age of Passive control group = 85.8 ± 5.1 *N* = 135	Intervention: Life Story Approach (a specific reminiscence program) Person delivering the intervention: Psychologist who had experience working with older adults and persons living with dementia or disabilities Format: individual sessions Length/duration: 2x/ week, 60 min, for 12 weeks Material: Photographs, recordings, and newspapers clippings to promote personal and shared memories were used		Intervention: Counselling sessions and informal social contacts (Active control) Person delivering the intervention: Psychologist who had experience working with older adults and persons living with dementia or disabilities Format: Individual sessions Length/duration: 2x/ week, 60 min, for 12 weeks Material: None Intervention: Unstructured social contact (Passive control) Person delivering the intervention: Psychologist who had experience working with older adults and persons living with dementia or disabilities Format: Individual sessions Length/duration: 2x/ week, 60 min, for 12 weeks Material: None
**Authors, Year, and Language of Publication**	**Study Design**	**Setting**	**Sample Characteristics and Sample Size**	**Experimental Intervention**		**Control or Comparison Intervention**
Haight et al., 2003 [27] (English)	Quasi-experimental design, with three arms (both caregiver and care receiver participate in Life Review, only caregiver participate in Life Review, and a no-treatment group)	At home	Diagnosed with Alzheimer’s Disease using the Functional Assessment Staging (FAST), and Global Deterioration Scale (GDS*); 40% male, 79% Caucasian, and 60% married; Age of sample not reported *N* = 44 (22 dyads of PWD and carer)	Intervention: Dyadic Life Review (both caregiver and care receiver participating in Life Review) Person delivering the intervention: a Reviewer (therapeutic listener), from the research team Format: one on one (caregiver and care receiver participate in Life Review separately, with two reviewers visiting their home Length/duration: 8 weeks of 1 h weekly visits, 2 weeks to open and close, and 6 weeks to review one’s life Material: Photos, props, and words were selected by patients themselves		Intervention: Life Review Person delivering the intervention: a Reviewer (therapeutic listener), from the research team Format: one on one (but only the caregiver participating in Life Review with a reviewer) Length/duration: 8 weeks of 1 h weekly visits, 2 weeks to open and close, and 6 weeks to review one’s life Material: Photos, props, and words were selected by patients themselves Intervention: Untreated control group Person delivering the intervention: not applicable Format: not applicable Length/duration: not applicable Material: None
**Authors, Year, and Language of Publication**	**Study Design**	**Setting**	**Sample Characteristics and Sample Size**	**Experimental Intervention**	**Control or Comparison Intervention**	
Hamann et al., 2000 [28] (English)	Quasi-experimental design with three arms- group with Alzheimer’s disease tested in a single session (AD group), healthy older adult controls tested in a single session (old group), healthy older adult controls tested within 2-week delay (old-delay. group)	Clinic	AD group: classified as having mild to moderate dementia, based on scores on MMSE, Age = 71.5 ± 2.4 Old group: scores on MMSE above 26 points (within normal range), Age = 72.2 ± 2.4 Old-Delay group: scores on MMSE above 26 points (within normal range), Age = 70.8 ± 2.8 *N* = 36	Intervention: AD group, viewing of emotionally arousing (both pleasant and unpleasant) photos Person delivering the intervention: Researcher/ experimenter Format: individual Length/duration: one-off, around a couple of hours Material: All groups shown 60 digitized color photographs (20 positive, 20 negative, 20 neutral) + 60 distractor photographs	Intervention: Old group, and Old-delay group, viewing of emotionally arousing photos Person delivering the intervention: Researcher/experimenter Format: individual Length/duration: one-off, around a couple of hours for Old-group; couple of hours, then again 2 weeks later for Old-delay group Material: All groups shown 60 digitized color photographs (20 positive, 20 negative, 20 neutral) + 60 distractor photographs	
Tamura et al., 2007 [30] (English)	Repeated measures design/ within-subjects design, no randomization: first trial showed washboard photo on PC, and fireplace photo on paper, second trial showed fireplace photo on PC, then washboard photo on paper	Nursing home or daycare	Diagnosed with dementia, with average MMSE score of 15.3 ± 7.8 points. Stable health a week before therapy, and ability to communicate with therapists and caregivers. Average age of 82.7 ± 6.0 years *N* = 6	Intervention: Reminiscence Therapy Person delivering the intervention: Therapist Format: individual sessions Length/duration: Two 5-min sessions, one week apart Material: photos of old-style Japanese fireplace and washboard on PC (personal computer)	Intervention: Reminiscence therapy Person delivering the intervention: Therapist Format: individual sessions Length/duration: Two 5-min sessions, one week apart Material: same photos as experimental group, but printed on paper	
Theijsmeijer et al., 2018 [25] (Dutch)	Two pilot RCT’s, with two arms each	Nursing home	Diagnosed with dementia, with GDS* score of 38.9 (in the first trial group), and 39.2 (second trial group); Average age of 87.4 ± 5.5 (first trial group), and 85.2 ± 5.9 (second trial group) *N* = 20	Intervention: Photo-intervention, using positive portraits (first trial), and person-oriented photos (second trial) Person delivering the intervention: not mentioned Format: individual sessions Length/duration: 30 min for both first and second trial (1 h in total), not mentioned if done in one day, or separate days Material: positive portraits (first trial), and person-oriented portraits (second trial)	Intervention: Photo-intervention using neutral portraits (first trial), and non-person-oriented photos (second trial) Person delivering the intervention: not mentioned Format: individual sessions Length/duration: 30 min for both first and second trial (1 h in total), not mentioned if done in one day, or separate days Material: neutral portraits (first trial), and non-person-oriented photos (second trial)	
**Authors, Year, and Language of Publication**	**Study Design**	**Setting**	**Sample Characteristics and Sample Size**	**Experimental Intervention**	**Control or Comparison Intervention**	
Tominari et al., 2021 [31] (English)	Open-label (no blinding), RCT with two arms	Elder care facilities and nursing homes	Diagnosed with dementia on the MMSE, with scores ranging from 22–26; Japanese speaking; no other psychological disorders or visual/auditory impairments; no previous experience with tablet-type devices; Average age 85.1 (intervention group), 87.0 (control group) *N* = 52	Intervention: Reminiscence Therapy using VR (Virtual Reality) technology Person delivering the intervention: certified nurse trained in reminiscence therapy Format: individual sessions Length/duration: one-on-one sessions, 30–45 min per session per week, for 8 weeks Material: digital photos of cultural artefacts belonging to time period that participants grew up (school classroom, shopping street, traditional Japanese house, etc.)	Intervention: Reminiscence Therapy using conventional paper-based photos Person delivering the intervention: certified nurse trained in reminiscence therapy Format: individual sessions Length/duration: one-on-one sessions, 30–45 min per session per week, for 8 weeks Material: paper-based photos of cultural artefacts belonging to time period that participants grew up (school classroom, shopping street, traditional Japanese house, etc.)	
Xu & Wang, 2020 [32] (English)	RCT with three arms	Care institution	Diagnosis of Alzheimer’s disease by their physicians and confirmed by researchers using the MMSE, no migraine or epilepsy, no issues with communication; Mean age of VR group = 76.7 ± 5.5; Photo group = 79.4 ± 2.0; Blank group 78.5 ± 3.4 *N* = 30	Intervention: VR-based reminiscence therapy group Person delivering the intervention: Researcher Format: individual sessions Length/duration: one-off session, 2 min for viewing, 5 min free-narrative recall Material: Photo of a Chinese rural cottage in the 1970’s was used, in VR environment	Intervention: Photo-based reminiscence therapy group Person delivering the intervention: Researcher Format: individual sessions Length/duration: one-off session; 2 min for viewing, 5 min free-narrative recall Material: color print photos of a Chinese rural cottage in the 1970’s was used Intervention: Blank group Person delivering the intervention: Researcher Format: individual sessions Length/duration: one-off session; 5 min free-narrative recall Material: no visual aids

**Table 2:** Outcome measures, type of photo and technology used, and results of included studies on social interaction and/or mood, and/or quality of life

Authors	Outcome Measures	Type of Photos used (Printed or Digitalized); Type of Technology used (if digitalized)	Results
Aşiret & Kapucu, 2016 [26]	• Mood: Geriatric Depression Scale (GDS) [57]	Printed photos	• A statistically significant (*p* < 0.01) decrease of 6.29 units in GDS score was found in individuals in the intervention group after reminiscence therapy
Azcurra, 2012 [29]	• Social Interaction: Social Engagement Scale (SES) [58] • Quality of Life: Self Reported Quality of Life Scale (SRQoL) [59]	Printed photos	• Statistically significant increase in intervention group between T2 and T0, and T1 and T0 (*p* < 0.01) • Statistically significant increase in intervention group between T0 and T1 and T1 and T2 (*p* < 0.01)
Haight et al., 2003 [27]	• Mood: Alzheimer’s Mood Scale	Printed photos	• Caregivers involved in intervention with care receiver rated mood of the care receivers (person with dementia) as significantly more improved (*p* < 0.04) compared to the other control groups (Care givers only group, and no treatment group)
Hamann et al., [28] 2000	• Mood: measured as emotional reactions to the photos, rated on a scale from 1 (least arousing) to 5 (most arousing)	Digitalized- PC/ color computer monitor	• Correlation between mean arousal ratings between AD group and Old + Old-Delayed group (healthy controls) were significantly large *r *[58] = .78, *p* < 0.0001, indicating that the AD group had ‘normal’ emotional responses to the photos
Tamura et al., 2007 [30]	• Social Interaction: measured as observed Voluntary Speech (positive, negative or no reply); and Talk (appropriate, inappropriate, or none) • Mood: measured by observing Facial Expression (pleasure, sadness, anger, indifference, or gazing)	Printed and Digitalized- PC (personal computer)	• No significant differences between methods reported • No significant difference between methods reported
Theijsmeijer et al., 2018 [25]	• Social Interaction: INTERACT observation scale [60] • Mood: Smiley Face Assessment Scale [23]	Printed photos	• No significant difference between groups reported, however a large positive effect size was detected for sub-scales- interaction with others (d = 0.25), and negative behaviour (d = 0.34), and medium positive effect size for speech (d = 0.31) and positive behaviour (d = 0.54), for the intervention group shown personalized photos • No significant differences between groups reported
Tominari et al., 2021 [31]	• Mood: Multidimensional Observation Scale for Elderly Subjects (MOSES) [61] • Quality of Life: PGC morale scale (evaluates subjective well-being of older adults [62]	Printed and Digitalized- VR panorama using tablet computer	• Degree of improvement in revised PGC morale scale score was significantly greater in the experimental group compared to the control group (*p* < 0.01) • No significant difference between groups reported
Xu & Wang, 2020 [32]	• Mood: Adapted Motivation questionnaire measuring interest, motivation, pleasantness, anxiety, security and fatigue (63, 64)	Printed and Digitalized- VR with stimulus displayed using HTC Vive Focus headset	• Significant difference reported in favor of experiencing pleasantness in the experimental group (*p* = 0.04)

#### Study setting and design

The majority of the studies found were set in nursing homes or daycare facilities [25, 26, 29–32] while one was set at home [27], and one study [28] was set in a clinic.

The study designs varied: Four studies were described as randomized controlled trials [25, 29, 31, 32], while the remaining four studies had a quasi-experimental design, described more fully in Table 1 [26–28, 30].

#### Psychosocial interventions using generic photos

Various kinds of psychosocial interventions were identified, and it was seen how each intervention utilized photos in the activities for people with dementia. The majority of the studies used reminiscence therapy [26, 30–32], where the individual or group members can share and reflect on past experiences by using props that prompt familiarity, whether it be in old music, songs, objects, or photographs, benefitting the participants as reminiscing can help them in finding or rekindling meaning and value in their personhood [48]. In the selected studies, it was noted that generic photos that were used in the activity included photos of old objects [26], photos of an old-style Japanese fireplace and washboard [30], photos corresponding to common cultural artifacts belonging to the time period where participants grew up, for example photos of classrooms, a traditional Japanese house, or a shopping street [31]; or photos of a 1970’s Chinese rural cottage [32].

Two studies [27, 29] used a variation of reminiscence therapy, namely Life Review, which aims to bring structure to the therapeutic work of reflecting on previous events and experiences across the life-span [49]. Life Review is an evaluative form of reminiscence [50, 51], where older adults benefit mentally from resolving past conflicts, allowing them to be more accepting of being in the late stages of their life [49]. Similar to the studies using conventional reminiscence therapy, Life Review also incorporated the use of photos, among other type of props, to elicit reminiscing and the retelling of significant life events [27, 29].

Two studies focused on using art-based interventions or activities for people with dementia [25, 28]. Psychosocial interventions that incorporate art for people with dementia were shown to benefit them by improving aspects of social interaction like communication and engagement [52, 53]. Hamann and colleagues [28] used photos from the International Affective Picture System [54] which is a standardized set of ‘target photos eliciting affect. Target photos were categorized as eliciting positive emotional arousal (e.g., infants or a romantic couple) or negative emotional arousal (e.g., famine victim or snake), while the rest was categorized as neutral (e.g., umbrella). A different set of positive, negative, and neutral photos were used as ‘distraction’ photos, in addition to the target photos. A mixture of these target and distraction photos were shown to participants and they were asked to rate each photo on an emotional arousal scale. Afterwards, their memory of the photos was tested [28].

Theijsmeijer and colleagues [25] tested different kinds of generic photos in two pilots, namely a) neutral portraits compared to positive portraits (of older and younger people from different cultural backgrounds) for the first condition, and b) generic photos that were person-oriented (meaning related to the person’s interests in their childhood and early adulthood) compared to generic photos that were not person-oriented (photos of cities, food, animals or landscapes) for the second condition. The photos were from a photo-database compiled by an artist who was also involved in the study. The photo-database had been used in the past to promote social interaction for people with dementia as well.

It is noted that the interventions all varied in terms of format (individual or group), length and duration of the interventions, with the shortest intervention lasting for 5 min [30] and the longest intervention lasting for an hour [27, 29] to a couple of hours [28], person delivering the intervention, and the materials used (refer to Table 1). Some interventions were delivered by therapists, psychologists or other professionals (like nurses) trained in the intervention [29–31], while others were delivered by members of the research team [26–28, 32]. It is also noted that most studies did not mention differences in the effects of the intervention with regards to level of cognitive impairment of the participants, except for one study, which used the Life Review [27]. In this study, the authors discussed two participants with a Global Deterioration scale (GDS) score [55] of 4 and 6 of which the participant with GDS 4 may have had more of her cognitive skills intact to work through the Life Review. The participant with GDS 6 experienced more agitation and anxiety due to resurfacing trauma while doing the Life Review. The authors hypothesized that this participant may have been able to talk about previous trauma during the Life Review, because the later stage of dementia negated the defense mechanisms that may have kept the trauma locked in previously [27].

#### Psychosocial interventions using technology

Four of the included studies incorporated technology in their interventions, namely a personal computer [28, 30], and VR (virtual reality) technology [31, 32]. Both studies using VR technology reported better outcomes for the group who viewed the photos using VR, compared to printed photos [31, 32], while the study comparing viewing photos on the PC versus printed photos reported no significant differences in outcomes, leading to the conclusion that it is feasible to conduct photo-based interventions digitally [30].

#### Outcomes measured

None of the included studies measured all three outcomes of interest (social interaction, mood, quality of life). Most of the studies measured mood of the person with dementia [25–28, 30–32]. A few measured social interaction [25, 29, 30], and a few measured quality of life [29, 31]. Most of the studies that measured the same outcomes used different measuring instruments, except for Tamura [30] and Theijsmeijer [25] who both used a variation of facial expression scales to measure mood of the person with dementia.

### Results on mood and/or social interaction and/or quality of life

Table 2 summarizes the quantitative results from the included studies, in relation to social interaction and/or mood and/or quality of life. In terms of social interaction, only one study reported positive, statistically significant outcomes on the intervention group versus the control group [29]. Theijsmeijer [25] reported no significant outcomes of their small, underpowered, pilot studies, however, they reported medium to large positive effect sizes for sub-scales measuring interaction with others, negative behaviour, speech, and positive behaviour for the intervention group that was shown personalized generic photos.

Four studies reported positive and statistically significant outcomes for mood in the intervention group [26, 27, 31, 32]. One study measured emotional reaction or arousal to photos, which was decided to be included in the mood outcome category [28]. This study aimed to explore whether emotional arousal would affect photo recall, and they found that despite their participants with dementia having similar emotional reactions to the photos as the controls, their memory and recall of the photos were still impaired [28]. Only one study found a statistically significant effect on the outcome quality of life, favoring the intervention group [29].

### Quality and weight-of-evidence assessment of included studies

Table 3 presents the quality assessment and weight of evidence of the included studies. Only two studies were found to have Good methodological quality [31, 32], while four studies were judged as having Fair methodological quality [25, 26, 28, 29]. Two studies were judged as having Poor methodological quality [27, 30] due to lack of reporting on randomization, having no blinding and no reported adherence to intervention protocols, or lack of valid and reliable measures used in the study. Despite these methodological limitations, one of the studies with Poor methodological quality turned out to have an overall rating of Fair, due to having characteristics relevant to the current review [30], such as the intervention using generic photos, and having integrated technology into the activity (PC). On the other hand, while the study of Hamann and colleagues [28] received a Fair rating for methodological quality, it received an overall rating of Low due to the relevance of its study characteristics to this review, namely that majority of the participants were healthy older adults. It was still decided to include this paper as it explicitly stated using generic photographs. The study by Haight and colleagues [27] also received an overall rating of Low, due to the photos not being explicitly described as generic, but was still included due to the very small number of studies available in the literature. Studies with quasi-experimental design were also included in the narrative synthesis due to the lack of good quality RCT’s incorporating generic photographs, as they were still informative and had relevance to this current review [26, 30].

**Table 3:** Quality and weight-of-evidence assessment of included studies

Authors	WoE A (Good, Fair, Poor)	WoE B (High, Fair, Low)	WoE C (High, Fair, Low)	WoE D (High, Fair, Low)
Aşiret & Kapucu, 2016 [26]	Fair	Fair	Fair	Fair
Azcurra, 2012 [29]	Fair	High	Fair	Fair
Haight et al., 2003 [27]	Poor	Fair	Low	Low
Hamann et al., 2000 [28]	Fair	Low	Low	Low
Tamura et al., 2007 [30]	Poor	Fair	High	Fair
Theijsmeijer et al., 2018 [25]	Fair	High	High	High
Tominari et al., 2021 [31]	Good	Fair	High	Fair
Xu & Wang, 2020 [32]	Good	High	High	High

*WoE* Weight of Evidence

## Discussion

This systematic review identified 8 papers that utilized generic photographs in psychosocial interventions for people with dementia to improve their social interaction and/or mood and/or quality of life. Using photographs as a form of art-based intervention has significant potential in providing activities for people with dementia and their carers that are easier to implement, less costly (compared to activities that require transportation like museum trips or buying materials for painting activities for example), and more culturally flexible, especially for people with dementia and their families in lower income countries, where there is more reliance on the informal carer due to lack of quality healthcare [1, 4, 7]. Photos of everyday objects, places or common events [26], might also provide a more relatable topic of conversation as it is less subjective and more open to personal interpretations.

The use of generic photographs specifically, still seems to be an uncommon tool when it comes to designing psychosocial interventions for people with dementia and their carers. Of the few studies found using generic photographs for example, it was noted that the chosen photos were still meant to be personal or to have meaning or relevance to the participants, as in the case of the person-oriented-photos in Theijsmeijer’s study [25], and photos of cultural artefacts relevant to the time period in which the participants grew up, in Tominari [31], Tamura [30] and Xu and Wang’s [32] studies. However, only Theijsmeijer’s study [25] made a comparison between the different kinds of generic photographs used, and indeed while no significant effects were found due to small sample size, some promising tendencies were found in favour of person-oriented photos. This supports the findings from Astell and colleagues [16] where the participants with dementia spoke more and shared more of their stories when viewing photos of generic annual events, compared to family photos. Personal and family photos may indeed feel more like a ‘test’ to the person with dementia, leading to frustration, or from a logistical perspective, may be more difficult to obtain from the person with dementia or the family members [16, 26, 29].

This review found that the most common type of psychosocial intervention studied that used generic photographs is reminiscence [26, 30–32].While reminiscence as a therapy in general has been covered extensively in literature, it seems that a lot of inconsistencies in the structure and methods of implementing it exist, in addition to it generally having small effect-sizes [34]. Activities based on reminiscence therapy, while aiming to have more structure, seem to involve a lot of time investment for training or implementation [27, 29]. The second type of psychosocial intervention that used generic photographs was in the form of art-viewing, which seemed to take less time and effort to implement [25]. Of note is the study of Tyack [17], which was not part of the final inclusions, but was the only other study that came up in the search results which also implemented a form of art-viewing activity (on a tablet computer), using generic photos of objects and paintings from museums. In general, it was noted that the included studies, despite sometimes having the same intervention type (i.e. reminiscence), all had a lot of variation in terms of key study characteristics like length and duration of the interventions, materials used, and also the person delivering the intervention (i.e. a clinical professional, or a member of the research team). This review focused on outcomes of social interaction and/or quality of life and/or mood. Social interaction or participation in social activities, is defined by INTERDEM as an important aspect of maintaining or enhancing social health for persons with dementia [6]. A qualitative study [56] found that meaningful social interactions improve quality of life for residents with dementia in nursing homes. Residents in this study reported that it was difficult to form relationships with their formal carers because the carers were too busy and focused on tasks, making the days of the residents long and lonely. The residents mentioned that they wished their days could be filled with conversations that could stimulate the mind. They look forward to visits of families, but when family connections are not as strong, residents also tend to look to their formal carers for companionship [56]. In another study, residents with dementia in the nursing home were found to have better mood, the more social interaction they had [57]. This was an important finding because it was observed that efforts residents made to interact with staff for example, were ignored, possibly leading to a decreased sense of agency which is fundamental for well-being of people with dementia [57, 58].

In the current review, none of the included studies measured all three outcomes of interest (social interaction, mood, quality of life), despite these outcomes being interwoven in the overall well-being of people with dementia. Despite limited studies, it was found that interventions that included generic photos had positive effects on the outcomes of interest. Most of the significant findings were in studies that measured mood outcomes [26, 27, 31, 32], followed by social interaction and quality of life [29], wherein improvements were found in favour of their interventions. While no significant effects were found in other studies, medium to large effects sizes were detected for social interaction in favour of an intervention group using person-oriented photos compared to non-person oriented photos [25]. It was shown that a variety of questionnaires and methods were used to measure these outcomes, and that only the use of facial expression scales to measure mood were found to be similar in some studies [25, 30].

The limited availability of studies focusing on psychosocial interventions using generic photos for people with dementia meant that some studies that were given an overall Low quality assessment rating were still included in the overall narrative synthesis [27, 28]. Out of the final eight included studies, only two achieved an overall quality assessment rating of High [25, 32], highlighting the need for more studies with better experimental designs.

Finally, it was found that at least half of the included studies incorporated the use of technology in their interventions, like personal computers and VR technology [28, 30–32]. This is at least a positive finding because it was concluded in a systematic literature review [18] that while most current studies using technology are small-scale, they have the potential to provide meaningful social activities for persons with dementia, and to reduce the pressure and strain on the carers by providing easier ways to interact and communicate, ultimately enhancing the quality of the relationship between person with dementia and carer. While only one study in this current review actually used digitalized generic photos in their intervention [28], the other studies at least provided good supporting evidence that using digitalized generic photos is the same, or is sometimes even better compared to conventional, printed photos [30–32]. Again of note is the study by Tyack and colleagues [17] who also used digital generic photos for art-viewing on a tablet computer and concluded that art interventions delivered through touch-screen devices can also be beneficial for the well-being of persons with dementia and their carers. The fast-paced development of technology and its increasing availability means that it can provide innovative solutions in addressing issues relating to dementia and ageing [59]. It may make interventions easier to implement and disseminate, a quality that can be especially helpful for people with dementia and their carers living in developing countries [7].

### Strengths and limitations of this review

This review was able to implement a systematic search strategy with the help of one of the authors (C.P.) who is an information specialist. This is the main strength of the review. The search strategy was discussed extensively in the review team to create search strings, and in addition MeSH terms, thesaurus terms and free terms were also used. Seven databases were included in the search, and relevant systematic reviews that came up in the search results were screened by J.T. for any additional studies that might fit the inclusion criteria. Reviewers were paired during independent title screening to help minimize bias. Quality assessment was also undertaken by two junior researchers, and then reviewed by a senior researcher. The review also did not exclude based on language, if the abstract was also available in English. Only one result in this review was in a language other than English [25], and an online translating website was used to translate Dutch to English.

As the review was limited to established databases, a number of studies that may have been applicable based on the review’s criteria but have been published in journals that are not included in the prominent databases may have been left out. This review also did not investigate grey literature.

Due to the limited number of results, some amount of flexibility had to be applied in terms of the inclusion criteria, as long as the reviewers who did the quality assessment all agreed. The results rated as having Low quality assessment ratings for example, may be lacking in some criteria, but still add relevant information to this review.

It should be noted that the final additional search update conducted in June 2023, to check if new relevant intervention studies were conducted between the first search and time of reviewing of the manuscript by the journal, was carried out by the first author (J.T.) only, because of time constraints. Although the same methods of inclusion and exclusion of studies were followed, and the selection process has taken place carefully, this can be seen as a limitation.

### Recommendations and implications for future research

Despite the potential of using generic photos in psychosocial activities for people with dementia [16, 25], this review found a very limited number of studies that evaluated interventions using generic photographs. While personal photos i.e. family photos, have mostly been used in reminiscence interventions [34], some of the studies [31, 32] included in this review showed that generic photos can still have relevance if personalized or chosen with the person in mind.

It is encouraging to see that in the limited, and often small-scale, studies, there are reports of positive effects of these interventions in social interaction and/or mood and/or quality of life. Future work in this area should therefore address the need for studies with better methodological quality (i.e. randomized controlled trials with larger sample sizes) and consider looking into the effects of interventions using generic photos on all of the three outcomes (social interaction, mood, quality of life) combined, because as mentioned in the discussion, these outcomes are interwoven aspects of the social health of people living with dementia [6]. While only quantitative studies were included in this review, it is noted that qualitative studies may also contain valuable insight and may be considered in future reviews, especially where the intention is to get insight into the mechanisms of impact of the use of generic photos in psychosocial interventions and conditions for implementing these interventions successfully.

Based on the findings of this review, exploring different kinds of activities that use generic photographs might be beneficial, as it was observed that activities based on reminiscence alone may take too much time in terms of training and implementation [27, 29]. Activities that use generic photos as the main tool may be easier to set-up in terms of acquiring the photos and designing social activities around it. Integrating technology to enhance psychosocial activities is also worth investigating further. This is based on this review’s findings that using technology to view digital photos is similar or in some cases more enjoyable compared to printed photos [30–32]. Technology is becoming more advanced and available, potentially making interventions easier to implement. This becomes especially relevant in the current times, where the known issue of social isolation of people with dementia, especially those living in nursing homes, is again being exacerbated due to the global Covid-19 pandemic [60–62].

## Conclusions

To our knowledge, this is the first systematic literature review that looked into psycho-social interventions that made use of generic photos (whether on their own or in combination with other materials in the intervention), and the effects of these interventions on social interaction and/or mood and/or quality of life of persons with dementia. The amount of relevant literature with good methodological quality is limited, so only a small sample of studies were explored in this review. It is therefore not possible to draw firm conclusions on the effectiveness of psychosocial interventions using generic photos. However, psychosocial interventions using generic photographs can be a promising area for future research, especially if explored in tandem with the use of technology (i.e. technology supported social activities like viewing digital photos on a tablet computer), allowing for easy accessibility, scalability, and personalization.

### Supplementary Information

**Additional file 1. **PRISMA 2020 checklist.

**Additional file 2. **PRISMA 2020 abstract checklist.

**Additional file 3.** Supplementary- Advanced search strings.

## Data Availability

Not applicable.

## Abbreviations

AD: Alzheimer’s Disease
APA PsycInfo: American Psychological Association, Database for behavioural and social sciences literature
CENTRAL: Cochrane repository for all reports of trials
Cinahl: Database of top nursing journals
DLB: Dementia with Lewy Bodies
DSM-IV: Fourth edition of the Diagnostic and Statistical Manual of Mental Disorders
EBSCO: Provider of research databases, e-journals, etc.
Embase: Database for medical literature
FAST: Functional Assessment Staging
GDS: Geriatric Depression Scale
GDS*: Global Deterioration Scale
HTC: High Tech Computer
INTERACT: Social interaction observation scale
INTERDEM: Early detection and timely INTERvention in DEMentia
MeSH: Medical subject headings
MMSE: Mini-Mental State Examination
MOSES: Multidimensional Observation Scale for Elderly Subjects
MRI: Magnetic resonance imaging
PICO: Population, intervention, comparison, outcome
PC: Personal Computer
PGC: Philadelphia Geriatric Center
PRISMA: Preferred reporting items for systematic reviews and meta-analyses
PubMed: Database for biomedical and life sciences literature
PWD: Person with dementia
RCT: Randomized controlled trial
Scopus: Bibliographic database for academic journal articles
SES: Social Engagement Scale
SRQoL: Self-Reported Quality of Life Scale
VR: Virtual reality
WHO: World Health Organization
WoE: Weight of Evidence
