# Supplementary material for: The effects of psychosocial interventions using generic photos on social interaction, mood and quality of life of persons with dementia: a systematic review
Source: BMC Geriatr. 2023 Sep 14;23:560. doi: 10.1186/s12877-023-04270-w (PMC10500875; doi:10.1186/s12877-023-04270-w)
Supplement: Supplementary file 3 — Additional file 3. Supplementary- Advanced search strings. [file 12877_2023_4270_MOESM3_ESM.docx]

Supplementary- Advanced search strings

|  | PubMed |  |
| --- | --- | --- |
| Search | Query | Results |
| #4 | Search: #1 AND #2 AND #3 Sort by: Most Recent | [307](https://pubmed.ncbi.nlm.nih.gov/?term=%231+AND+%232+AND+%233&sort=date) |
| #3 | Search: "Treatment Outcome"[Mesh] OR outcome*[tiab] OR effect*[tiab] OR "qualitative"[tiab] OR "evaluation*"[tiab] OR "mixed-method*"[tiab] OR "quantitative"[tiab] Sort by: Most Recent | [11,852,767](https://pubmed.ncbi.nlm.nih.gov/?term=%22Treatment+Outcome%22%5BMesh%5D+OR+outcome%2A%5Btiab%5D+OR+effect%2A%5Btiab%5D+OR+%E2%80%9Cqualitative%E2%80%9D%5Btiab%5D+OR+%E2%80%9Cevaluation%2A%E2%80%9D%5Btiab%5D+OR+%E2%80%9Cmixed-method%2A%E2%80%9D%5Btiab%5D+OR+%E2%80%9Cquantitative%E2%80%9D%5Btiab%5D++&sort=date) |
| #2 | Search: "Photography"[Mesh:NoExp] OR photo[tiab] OR photograph[tiab] OR photographs[tiab] OR photos[tiab] OR "photo’s"[tiab] OR "art picture*"[tiab] OR "art image*"[tiab] OR photography[tiab] OR portrait[tiab] OR portraits[tiab] Sort by: Most Recent | [118,985](https://pubmed.ncbi.nlm.nih.gov/?term=%22Photography%22%5BMesh%3ANoExp%5D+OR+photo%5Btiab%5D+OR+photograph%5Btiab%5D+OR+photographs%5Btiab%5D+OR+photos%5Btiab%5D+OR+%E2%80%9Cphoto%E2%80%99s%E2%80%9D%5Btiab%5D+OR+%E2%80%9Cart+picture%2A%E2%80%9D%5Btiab%5D+OR+%E2%80%9Cart+image%2A%E2%80%9D%5Btiab%5D+OR+photography%5Btiab%5D+OR+portrait%5Btiab%5D+OR+portraits%5Btiab%5D&sort=date) |
| #1 | Search: "Dementia"[Mesh] OR "Cognitive Dysfunction"[Mesh] OR dement*[tiab] OR Alzheimer*[tiab] OR "cognitive impairment*"[tiab] OR "cognitive decline*"[tiab] OR "cognitive dysfunction"[tiab] OR MCI[tiab] Sort by: Most Recent | [399,938](https://pubmed.ncbi.nlm.nih.gov/?term=%22Dementia%22%5BMesh%5D+OR+%22Cognitive+Dysfunction%22%5BMesh%5D+OR+dement%2A%5Btiab%5D+OR+Alzheimer%2A%5Btiab%5D+OR+%E2%80%9Ccognitive+impairment%2A%E2%80%9D%5Btiab%5D+OR+%E2%80%9Ccognitive+decline%2A%E2%80%9D%5Btiab%5D+OR+%E2%80%9Ccognitive+dysfunction%E2%80%9D%5Btiab%5D+OR+MCI%5Btiab%5D&sort=date) |

Key: [Mesh]= medical subject heading, [tiab] = title, abstract, author supplied keywords.

|  | Embase (Embase.com) |  |
| --- | --- | --- |
| No. | Query | Results |
| #5 | #4 AND ('article'/it OR 'article in press'/it OR 'chapter'/it OR 'conference paper'/it OR 'conference review'/it OR 'editorial'/it OR 'note'/it OR 'review'/it) | 359 |
| #4 | #1 AND #2 AND #3 | 546 |
| #3 | 'treatment outcome'/exp OR outcome*:ab,ti,kw OR effect*:ab,ti,kw OR 'qualitative':ab,ti,kw OR 'evaluation*':ab,ti,kw OR 'mixed-method*':ab,ti,kw OR 'quantitative':ab,ti,kw | 15,358,864 |
| #2 | 'photography'/de OR photo:ab,ti,kw OR photograph:ab,ti,kw OR photographs:ab,ti,kw OR photos:ab,ti,kw OR 'photo-s':ab,ti,kw OR 'art picture*':ab,ti,kw OR 'art image*':ab,ti,kw OR photography:ab,ti,kw OR portrait:ab,ti,kw OR portraits:ab,ti,kw | 142,511 |
| #1 | 'dementia'/exp OR 'mild cognitive impairment'/exp OR dement*:ab,ti,kw OR alzheimer*:ab,ti,kw OR 'cognitive impairment*':ab,ti,kw OR 'cognitive decline*':ab,ti,kw OR 'cognitive dysfunction':ab,ti,kw OR mci:ab,ti,kw | 632,541 |
|  |  |  |

Key: Ab, ti, kw searches in abstract, title and author supplied keywords, /exp searches Emtree preferred indexing term, it=publication type

|  | APA PsycInfo (EBSCO) |  |
| --- | --- | --- |
| # | Query | Results |
| S4 | S1 AND S2 AND S3 | 165 |
| S3 | DE "Psychotherapeutic Outcomes" OR DE "Side Effects (Treatment)" OR DE "Treatment Outcomes" OR TI(outcome* OR effect* OR “qualitative” OR “evaluation*” OR “mixed-method*” OR “quantitative”) OR AB(outcome* OR effect* OR “qualitative” OR “evaluation*” OR “mixed-method*” OR “quantitative”) OR KW(outcome* OR effect* OR “qualitative” OR “evaluation*” OR “mixed-method*” OR “quantitative”) | 2,203,633 |
| S2 | DE "Photographs" OR DE "Digital Images" OR DE "Photographic Art" OR TI(photo OR photograph OR photographs OR photos “photo’s” OR “art picture*” OR “art image*” OR “photography” OR portrait OR portraits) OR AB(photo OR photograph OR photographs OR photos “photo’s” OR “art picture*” OR “art image*” OR “photography” OR portrait OR portraits) OR KW(photo OR photograph OR photographs OR photos “photo’s” OR “art picture*” OR “art image*” OR “photography” OR portrait OR portraits) | 26,213 |
| S1 | DE "Neurocognitive Disorders" OR DE "AIDS Dementia Complex" OR DE "Dementia with Lewy Bodies" OR DE "Pseudodementia" OR DE "Semantic Dementia" OR DE "Vascular Dementia" OR DE "Cognitive Impairment" OR DE "Creutzfeldt Jakob Syndrome" OR DE "Mild Cognitive Impairment" OR DE "Picks Disease" OR DE "Senile Dementia" OR DE "Dementia" OR DE "Senile Psychosis" OR DE "Alzheimer's Disease" OR DE "Presenile Dementia" OR DE "Cognitive Aging" OR DE "Cognitive Impairment" OR TI(dement* OR Alzheimer* OR “cognitive impairment*” OR “cognitive decline*” OR “cognitive dysfunction”) OR AB(dement* OR Alzheimer* OR “cognitive impairment*” OR “cognitive decline*” OR “cognitive dysfunction”) OR KW(dement* OR Alzheimer* OR “cognitive impairment*” OR “cognitive decline*” OR “cognitive dysfunction”) | 173,132 |

Key: DE= Descriptors,TI= title, AB= abstract and KW= Searches for keywords in the uncontrolled content description of the document

|  | **Cinahl (EBSCO)** |  |
| --- | --- | --- |
| **#** | **Query** | **Results** |
| S4 | S1 AND S2 AND S3 | 131 |
| S3 | (MH "Treatment Outcomes+") OR TI(outcome* OR effect* OR “qualitative” OR “evaluation*” OR “mixed-method*” OR “quantitative”) OR AB(outcome* OR effect* OR “qualitative” OR “evaluation*” OR “mixed-method*” OR “quantitative”) OR KW(outcome* OR effect* OR “qualitative” OR “evaluation*” OR “mixed-method*” OR “quantitative”) | 2,466,541 |
| S2 | (MH "Photography") OR (MH "Digital Imaging") OR TI(photo OR photograph OR photographs OR photos OR “photo’s” OR “art picture*” OR “art image*” OR photography OR portrait OR portraits) OR AB(photo OR photograph OR photographs OR photos OR “photo’s” OR “art picture*” OR “art image*” OR photography OR portrait OR portraits) OR KW(photo OR photograph OR photographs OR photos OR “photo’s” OR “art picture*” OR “art image*” OR photography OR portrait OR portraits) | 28,737 |
| S1 | (MH "Dementia+") OR (MH "Creutzfeldt-Jakob Syndrome+") OR (MH "Dementia, Vascular+") OR (MH "Frontotemporal Lobar Degeneration+") OR (MH "Mild Cognitive Impairment") OR TI(dement* OR Alzheimer* OR “cognitive impairment*” OR “cognitive decline*” OR “cognitive dysfunction” OR MCI) OR AB(dement* OR Alzheimer* OR “cognitive impairment*” OR “cognitive decline*” OR “cognitive dysfunction” OR MCI) OR KW(dement* OR Alzheimer* OR “cognitive impairment*” OR “cognitive decline*” OR “cognitive dysfunction” OR MCI) | 136,871 |

Key: MH= Medical Headings,TI= title, AB= abstract and SU= Searches for keywords in the uncontrolled content description of the document

|  | **Web of Science (Core collection) - Clarivate** |  |
| --- | --- | --- |
| History Count | Search Terms | Results |
| 4 | #1 AND #2 AND #3 | 410 |
| 3 | TS=(outcome* OR effect* OR “qualitative” OR “evaluation*” OR “mixed-method*” OR “quantitative”) | 19,076,316 |
| 2 | TS=(photo OR photograph OR photographs OR photos OR “photo’s” OR “art picture*” OR “art image*” OR photography OR portrait OR portraits) | 368,875 |
| 1 | TS=(dement* OR Alzheimer* OR “cognitive impairment*” OR “cognitive decline*” OR “cognitive dysfunction” OR MCI) | 487,765 |

Key: TS = topic, which includes title, abstract, author keywords and Web of Science Keywords Plus.

|  | **Scopus (Elsevier)** |  |
| --- | --- | --- |
| **Nr.** | **Query** | Results |
| 4 | #1 AND #2 AND #3 | 580 |
| 3 | TITLE-ABS-KEY ( outcome*  OR  effect*  OR  "qualitative"  OR  "evaluation*"  OR  "mixed-method*"  OR  "quantitative" ) | 29,120,981 |
| 2 | TITLE-ABS-KEY ( photo  OR  photograph  OR  photographs  OR  photos  OR  "photo's"  OR  "art picture*"  OR  "art image*"  OR  photography  OR  portrait  OR  portraits ) | 629,406 |
| 1 | TITLE-ABS-KEY ( dement*  OR  alzheimer*  OR  "cognitive impairment*"  OR  "cognitive decline*"  OR  "cognitive dysfunction"  OR  mci ) | 519,492 |

Key: ABS, TITLE, KEY searches in abstract, title and author supplied keywords

|  | Cochrane Database of Systematic Reviews + CENTRAL (Wiley) |  |
| --- | --- | --- |
| ID | Search | Hits |
| #6 | #4 AND CENTRAL | 81 |
| #5 | #4 AND Cochrane reviews | 2 |
| #4 | #1 AND #2 AND #3 | 88 |
| #3 | (outcome* OR effect* OR qualitative OR evaluation* OR “mixed-method” OR “mixed methods” OR quantitative):ab,ti,kw | 1347228 |
| #2 | (photo OR photograph OR photographs OR photos OR “photo’s” OR “art picture” OR “art pictures” OR “art image” OR “art images” OR photography OR portrait OR portraits):ab,ti,kw | 8554 |
| #1 | (dement* OR Alzheimer* OR “cognitive impairment” OR “cognitive decline” OR “cognitive dysfunction” OR MCI):ab,ti,kw | 30735 |

Key: ti,ab,kw searches in title, abstract and author supplied keywords
